# A novel graph convolutional neural network for predicting interaction sites on protein kinase inhibitors in phosphorylation

**DOI:** 10.1038/s41598-021-04230-7

**Published:** 2022-01-07

**Authors:** Feiqi Wang, Yun-Ti Chen, Jinn-Moon Yang, Tatsuya Akutsu

**Affiliations:** 1 Bioinformatics Center, Insititute for Chemical Research, Kyoto University, Gokasho, Uji, Kyoto 611-0011 Japan; 2 Institute of Bioinformatics and Systems Biology, National Yang Ming Chiao Tung University, Hsinchu, 300 Taiwan; 3 Department of Biological Science and Technology, National Yang Ming Chiao Tung University, Hsinchu, 300 Taiwan; 4 Bioinformatics Center, Insititute for Chemical Research, Kyoto University, Gokasho, Uji, Kyoto 611-0011 Japan

**Keywords:** Computational biology and bioinformatics, Computational models, Machine learning, Protein analysis

## Abstract

Protein kinase-inhibitor interactions are key to the phosphorylation of proteins involved in cell proliferation, differentiation, and apoptosis, which shows the importance of binding mechanism research and kinase inhibitor design. In this study, a novel machine learning module (i.e., the WL Box) was designed and assembled to the Prediction of Interaction Sites of Protein Kinase Inhibitors (PISPKI) model, which is a graph convolutional neural network (GCN) to predict the interaction sites of protein kinase inhibitors. The WL Box is a novel module based on the well-known Weisfeiler-Lehman algorithm, which assembles multiple switch weights to effectively compute graph features. The PISPKI model was evaluated by testing with shuffled datasets and ablation analysis using 11 kinase classes. The accuracy of the PISPKI model with the shuffled datasets varied from 83 to 86%, demonstrating superior performance compared to two baseline models. The effectiveness of the model was confirmed by testing with shuffled datasets. Furthermore, the performance of each component of the model was analyzed via the ablation study, which demonstrated that the WL Box module was critical. The code is available at https://github.com/feiqiwang/PISPKI.

## Introduction

Phosphorylation of proteins, which is central to various biological processes and the regulation of most aspects of cell functions^1^, is a common but complex post-translational modification to modulate cell proliferation^2^, differentiation^3^, and apoptosis^4^. Many studies about protein post-translational modification have effectively taken the biology field forward by using machine learning methods^5,6^. A protein kinase is a phosphotransferase enzyme that catalyzes the transfer of phosphate ($\hbox {PO}_{4}^{3-}$) groups donated by high-energy adenosine triphosphate (ATP) molecules to specific residues in order to regulate activities of proteins^7–12^. Because phosphorylation is an important biochemical process, protein kinases have been investigated as potential therapeutic targets^13–17^. In addition, kinase inhibitors block the activities of kinases and are vital to inhibit the addition of phosphate groups to the target protein^18^. Here, exploring the binding mechanism plays a crucial role on kinase inhibitor design. Many studies on the development of molecular drugs have focused on protein kinase inhibitors for the treatment of infectious diseases^19^ and cancers^20^.

During the process of protein phosphorylation, the $\gamma$-phosphate group of the ATP molecule is replaced by a hydroxide ion from water that is hydrolyzed to an inorganic phosphate ion existing in the environment^21^. Afterward, protein kinases transport the inorganic phosphate ions to the residues of the protein substrates^22^, which are typically serine, threonine, or tyrosine residues^23^. Based on the specific phosphorylated residue, these molecules are classified as serine/threonine, tyrosine-specific, histidine-specific, and aspartyl/glutamyl protein kinases^24^. Although there exist various classes of protein kinases, the characteristics of members of the same class are homologous^25,26^. However, protein kinases can be incorporated into protein-ligand complexes that bind to molecular inhibitors^27^ that block the transportation process^28^. Kinase inhibitors interact with protein kinase residues via electrostatic forces, hydrogen bonding, and van der Waals forces at specific interaction sites. We define those atoms that have interactions with residues from protein kinases as the interaction sites for inhibitors in this research.

Bioinformatics is a versatile tool to research complicated biological processes, and machine learning continues to gain popularity for the development of tools to analyze biological data. A graph convolutional neural (GCN) network is a recently developed neural network to directly operate and analyze graphic structures and has been widely applied for analysis of protein-ligand complexes, structure-embedded graph representation^29^, structure-based virtual screening^30^, prediction of binding affinity^31,32^, and prediction of binding residues^34^. Moreover, many novel algorithms have been proposed for solving specific biological issues in recent years^33^. Although most previous studies have focused on issues with protein-ligand complexes, to our knowledge, there is no previous study on the prediction of the interaction sites of inhibitor molecules based on known protein kinase-ligand complexes. As compared with affinity prediction, the prediction of interaction sites with a GCN network is more intuitive, allowing for the collection of the features of protein kinase inhibitors for designing more effective drug design. Here, a novel machine learning module, the Weisfeiler–Lehman (WL) Box, was designed and a GCN network with WL Boxes was developed as a tool to predict the interaction sites of different classes of protein kinase inhibitors. The WL Box is based on an algorithm proposed in 1968 by Weisfeiler and Lehman to solve the *graph isomorphism problem*^35^. To the best of our knowledge, this is the first application of a GCN network to predict the interaction sites of protein kinase inhibitors. The result confirmed that the WL Box is an effective tool for the analysis of protein kinase inhibitors and drug prediction studies.

## Results

### Experiment

#### Database and datasets

Protein-ligand complexes and interaction sites were collected from the *sc-PDB* three-dimensional database of ligandable binding sites^36^ and grouped by protein UniProt identifications from the *Protein Data Bank*^37^. In total, 1,064 protein-ligand complexes datasets of 22 protein kinases were extracted and categorized into 11 corresponding kinase classes as shown in Supplementary Information A. A program was developed to convert the *mol2 file* to a model input file consisting of the feature matrix $F\in \{0,1\}^{35\times N}$ and the structure adjacency matrix $S\in \{0,1,2,3,4\}^{N\times N}$ for each inhibitor molecule consisting of *N* atoms from the protein-ligand complex. According to the *mol2* format, there are 35 atom types and eight bond types. The categorical features of every atom were *one-hot* encoded as a *color* label and aligned with the feature matrix *F*. The bond defined as *single*, *triple*, *dummy*, *unknown*, and *not connected* were classified as TYPE 1, a *double* bond as TYPE 2, an *amide* bond as TYPE 3, and an *aromatic* bond as TYPE 4. The TYPE 1 category consists of five bond types (i.e., *single*, *triple*, *dummy*, *unknown*, and *not connected*) in the dataset. Here, the *single* bond is the most common bond type, and the remaining four bond types are rare. The structure adjacency matrix *S* is a record of the connection relationships between two atoms and their corresponding BOND TYPE of the inhibitor molecule. TYPE 0 can be used for any two atoms with bond types that are not mentioned in the the *mol2 file*.

#### Setup

An individual prediction model was established for each class of kinases. An inhibitor molecule with *N* atoms provides *N* data pairs (*F*, *S*) by assigning a specific mark to the label of each atom of feature matrix *F*. If a marked atom binds with a residue of the kinase, the corresponding output assigned a value of 1, otherwise, 0. The binding types between atoms and residues were ignored, as the binding state was the focus of this study. Each of the original datasets was expanded to the one with at least 2,560 positive and 2,560 negative samples by using the method described in “Dataset expander program” section, and the resulting expanded rates are shown in the last column of Table 2. The dataset was randomly split into three parts: one tenth positive/one tenth negative datasets into the test dataset, one tenth positive/one tenth negative datasets into the validation dataset, and rest of datasets into the training dataset, where three datasets were totally non-overlapping. Training datasets were used to train PISPKI models of each kinase class for several epochs, and models were evaluated by validation datasets at each epoch after training. Furthermore, *bootstrapping* was applied to the training and validation datasets to uniformly assign samples at each epoch. The program was developed with *PyTorch*^38^. As shown by the model setup in Table 1, *early-stopping*^39^ was set to 5 epochs to avoid overfitting issues and the accuracy of the sixth to last validation was recorded. After training was completed, the model was evaluated with the testing dataset.

**Table 1:** Model setup.

Components	Parameters
WL Box 1	3 layers $\times$ 3 time steps
WL Box 2	3 layers $\times$ 3 time steps
Conv-layer 1	1 input channel
	2 output channel
	3 $\times$ 3 kernels
Conv-layer 2	2 input channel
	5 output channel
	3 $\times$ 3 kernels
SPP cofficient	feature matrix: 10
	Structure matrix: 3
Dense layers	2000 neurons $\times$ 5 layers
Activation function	Leaky ReLU
Ealry stopping	5 epochs
Dropout	0.05
Turn size	$2048 \times 2$(Training dataset)
	$256 \times 2$(Validation dataset)
Batch size	16

#### Noise elimination

Multiple protein-ligand complexes consisted of the same protein (kinase) and ligand (inhibitor), but with different interaction sites. However, unique confusing events can occur, such as the existence of a kinase inhibitor with two *crystal structures* ($\alpha$ and $\beta$) and an atom of the inhibitor that binds with a residue of the kinase of *crystal structure* $\alpha$ but does not bond with any residue of *crystal structure* $\beta$.

To eliminate this type of noisy data, the notation ${\mathcal {B}}_I^{(K)}$ was used to denote inhibitor molecule *I* having *M* crystal structures on kinase *K* to represent a set consisting of all atoms binding with *K*. With $B^{(K,I)}_m$ designating a binding atom set for one of the crystal structures composed of inhibitor *I* and kinase *K*, the following definition is obtained:

$$\begin{aligned} {\mathcal {B}}_I^{(K)}=\bigcup _{m=1}^MB^{(K,I)}_m. \end{aligned}$$

Then, for atom *i* of the inhibitor molecule *I*, the interaction state $Y^{(K,I)}_i$ for kinase *K* is determined by

$$\begin{aligned} Y^{(K,I)}_i=\left\{ \begin{array}{rl} 1, &{}\quad i\in {\mathcal {B}}_I^{(K)},\\ 0, &{}\quad otherwise. \end{array} \right. \end{aligned}$$

### Dataset expander program

**Figure 1:**
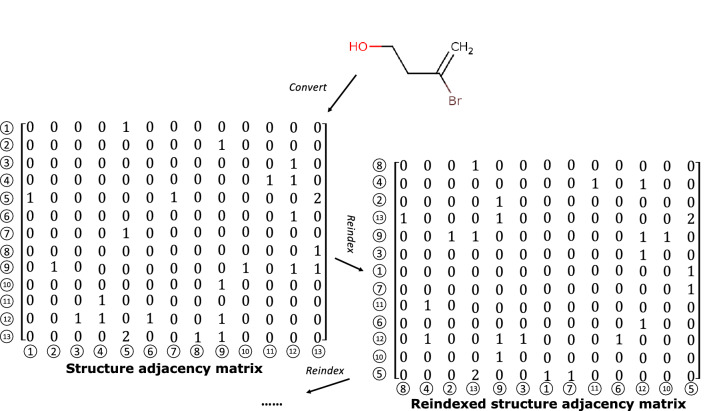
An example of the expander program.

Due to the limited number of original datasets (Supplementary Information A Table S2), a dataset expander algorithm was developed inspired by the expansion method widely applied with image recognition datasets. A *seed* is randomly assigned to the *reindex* *rows* or *columns* of matrices, and the *reindex* operation $\Psi$ does not change the structure of the inhibitor molecule but creates a different input data pair. An example of the structure adjacency matrix expansion is shown in Fig. 1. Each input pair (*F*, *S*) from the original dataset is modified to a new pair utilizing the same *seeds* for *F* and *S* while maintaining the same output *Y* as follows:

$$\begin{aligned} \left\{ \begin{array}{rl} F' &{} =\Psi _{r,c}(F,seed)\\ S' &{} =\Psi _{r}(S,seed)\\ Y' &{} =Y \end{array} \right. \end{aligned}$$

where $F'$, $S'$ and $Y'$ are the created feature matrix, structure adjacency matrix, and output, respectively. In addition, $\Psi _{r,c}$ indicates that the *reindex* operation was applied to both *rows* and *columns*, whereas $\Psi _r$ indicates application to rows only. By utilizing different *seeds* with an original pair, multiple different sample pairs can be obtained up to $P_N^N=N!$, where *N* is the atom number of the inhibitor molecule. The batch process method of the expander program is shown in Algorithm 1. As mentioned in “Experiment” section, original datasets transformed from the *mol2* format were collected with arrays of atoms in a particular order. The dataset expander program can also potentially support the model to improve compatibility with datasets collected from formats other than *mol2*.

**Table:** 

**Algorithm 1** Dataset expander program	
**Input**: Orignal feature dataset *F*; original structure dataset *S*; expansion rate *r*	
**Output**: Enlarged feature dataset $F_e$; enlarged structure dataset $S_e$	
1: original dataset length $l \leftarrow len(F)$	
2: random seed $seed \leftarrow random()$	
3: **for** $x=1$ to $l\times r$ **do**	
4: new feature matrix $f \leftarrow reindex_{row}(F[x\mod l],seed)$	
5: $f\leftarrow reindex_{column}(f,seed)$	
6: $F_e.append(f)$	
7: $S_e.append(reindex_{row}(S[x\mod l],seed))$	
8: **if** $x\mod 10==0$ **then**	
9: $seed \leftarrow random()$	
10: **end if**	
11: **end for**	
12: **return** $F_e$, $S_e$	

### Benchmark experiment

The performance of the proposed PISPKI model was comprehensively evaluated by comparison with Support Vector Machine (SVM) and Convolutional Neural Network (Conv-Net) models as baselines, where the SVM model applies the *radial basis function* kernel and Conv-Net has a traditional architecture consisting of two convolutional layers and a fully connected layer. Feature matrices with *zero-padding* were used as input for the baseline models. The highest accuracy of 10 repeated experiments was recorded. Comparison of the proposed PISPKI model and the two baseline models is shown in Table 2.

The accuracy of the PISPKI model to predict whether an atom from an inhibitor molecule is an interaction site or not mostly ranged from 83 to 86% for the different kinase classes, which was notably better than that of the two baseline models. In addition, both the Conv-Net and SVM models were unstable with different datasets of kinase classes, whereas the proposed model was not. Although the accuracy for the *Circadian clock protein kinase* was high, the prediction accuracy of the model is not necessarily high because the corresponding dataset contained only 16 protein-ligand complexes.

However, the expansion rate has no effect on the prediction of the interaction site, with the exception of extreme situations, such as the *Circadian clock protein kinase* mentioned above. Nonetheless, the performance of the model can be improved by applying a small number of expansion operations, as the accuracies of datasets with expansion rates of less than 3, such as the *mitogen-activated protein kinase*, *tyrosine-protein kinase*, *serine/threonine-protein kinase*, and *cyclin-dependent kinase*, are stable at about 86%.

**Table 2:** Comparison of the validation and test (%) performance of different models.

Kinase	Number of PLC*	Subclass	${N}_{{max}}$	Conv-Net (%)	SVM (%)	PISPKI(%)		Expansion rate (p/n)
						Validation	Test	
3-Phosphoinositide-dependent protein kinase	41	1	73	79.0	74.0	84.0	84.7	8/3
Aurora kinase	58	1	81	56.7	70.0	79.1	80.8	10/3
Circadian clock protein kinase	16	1	44	50.0	85.0	93.0	91.5	190/89
Cyclin-dependent kinase	280	1	67	76.0	68.3	86.7	85.1	2/1
Death-associated protein kinase	28	1	62	68.3	74.0	80.9	83.5	27/11
Dual specificity mitogen-activated protein kinase kinase	24	1	58	71.7	63.0	84.0	81.9	8/5
Glucokinase	20	1	55	78.3	75.0	85.5	85.5	25/9
Glycogen synthase kinase	40	1	74	61.7	67.0	84.4	85.5	11/3
Serine/threonine-protein kinase	197	4	69	63.3	67.3	85.4	85.0	2/1
Tyrosine-protein kinase	99	5	93	56.5	65.9	84.4	86.7	3/1
Proto-oncogene tyrosine-protein kinase	17	1	76	73.3	65.0	83.6	79.5	180/60
Mitogen-activated protein kinase	244	4	87	78.8	64.9	86.9	87.5	1/1

### Performance evaluation with shuffled datasets

**Figure 2:**
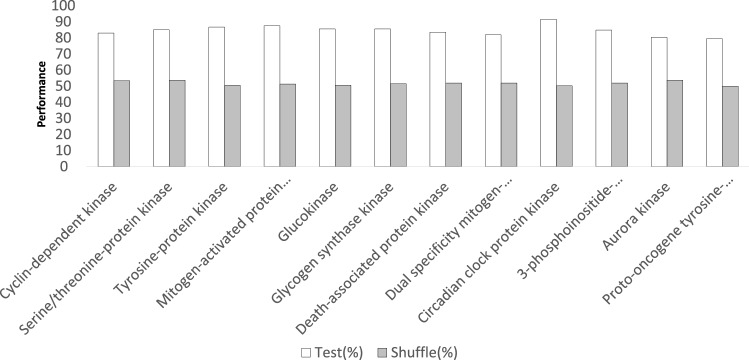
Comparison of the performance of the shuffled and testing datasets.

The effectiveness of the PISPKI model was further assessed with shuffled datasets. Due to the limited number, portions of the validation datasets were randomly extracted and the interaction sites were shuffled to create shuffled datasets. Consider two cases: (1) the PISPKI model could still predict the interaction sites of shuffled datasets with an accuracy equal to or greater than that of the testing datasets; and (2) the model is not compatible with shuffled datasets or the accuracy is obviously degraded. The baseline accuracy was set to 50% to denote the state “cannot work”, as such a situation is a binary classification issue. *Case (1)* suggests the model is compatible with both correct and incorrect data, indicating a problematic state, whereas *case (2)* confirmed the effectiveness of the model. The performances of the shuffled and testing datasets for each kinase class are compared in Fig. 2. The PISPKI model is incompatible with shuffled datasets, thereby validating its effectiveness.

### Ablation study

**Figure 3:**
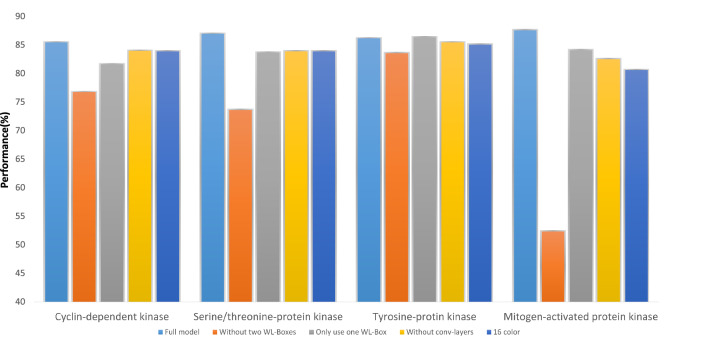
Ablation analysis.

To ensure and discuss the necessity of each part of the PISPKI model, an ablation experiment was designed in which the performance of the model was assessed by removing components. In the experiment, only four typical kinase class datasets with expansion rates of less than 3 were collected. Then, (1) two WL Boxes; (2) one WL Box; (3) and the Conv-layers were abandoned, and (4) 35 atom subtypes were merged into 16 *colors* by combining the same types of chemical elements to successively construct four incomplete models, which are illustrated in Supplementary Information B. The performance of the incomplete models was compared to that of the PISPKI model (Fig. 3).

The performance of the PISPKI model was significantly compromised by removing two WL Boxes from most datasets, which obviously decreased the accuracy. In addition, the model was incompatible with the *mitogen-activated protein kinase* dataset, thereby confirming that the WL Box is the core of the PISPKI model. As shown in the third column of each dataset in the figure, reducing the number of WL Boxes to one had very limited influence on the model. However, compared with the full model, the performance of the truncated model was improved by adding extra WL Boxes. The convolutional layers seem to be an insignificant component of in most datasets, which still suggests potential advantages. Notably, the convolutional layers only process original structures and feature label information as mentioned in “Method” section. Although the WL Boxes process the feature and structure information more exquisitely, the original information processed by the convolutional layers facilitates inference of the interaction sites more accurately in complicated cases. In the last ablation experiment, the necessity of feature richness, which represents the quality of each feature, was tested. For this evaluation, datasets from *mol2* files were collected, which encoded atoms in the *SYBYL* format that were further divided into 35 subtypes (*color*) and combined into 16 types (*color*) based on chemical elements as illustrated in Supplementary Information B Fig. S1(e). By the combination operation, the performance of the model with the different datasets decreased by various degrees. The results not only highlight the importance of *SYBYL* atom types but also serve as a reminder that the performance of the PISPKI model can be improved by enhancing feature richness.

In the ablation experiments, incomplete models were applied to examine differences in performance loss observed from the datasets. The effects on the *cyclin-dependent kinase* and *serine/threonine-protein kinase* datasets were very limited by applying incomplete models. However, the *tyrosine-protein kinase* and *mitogen-activated protein kinase* datasets had extremely low and high impacts, respectively. As mentioned above, the PISPKI model aims to solve the issue with interaction site prediction. However, there were multiple different sub-issues due to the kinase class, which is another reason why the accuracy of the baseline models was extremely variable with different kinase class datasets (Table 2).

## Discussion

In this study, a novel machine learning model (i.e., WL algorithm-based GCN network) was designed and developed to predict interaction sites of protein inhibitors in phosphorylation. The accuracy of the model was consistently 83–86%, which can be greatly improved by applying datasets with low expansion rates compared to the two baseline models. The model performance can be improved by the addition of feature richness. At present, features are transformed from inhibitor molecules based on *SYBYL* atom types, which contain more information than chemical elements. More information about atoms can be collected to enhance the richness of features such as *radius*, *atomic mass*, *formal charge*, and *aromaticity*. In addition, the protein kinase residue information should also be used as input to the model. Furthermore, the limitation of datasets effects the model performance. This research was not only limited by input feature richness but also by the small number of datasets because there have been relatively few investigations to identify the interaction sites of inhibitor molecules. The spatial pyramid pooling (SPP) module facilitated compatibility of the model with inhibitor molecules having different number of atoms. Furthermore, the importance of the WL Box was confirmed by the ablation study (“Ablation study” section), which showed that the addition of multiple WL Boxes can enhance performance. Although applying a complicated model on a simple issue is not recommended owing to potential performance degradation because of excess trainable parameters, the PISPKI model can predict most interaction sites and solve other complicated biology issues. Hence, stable model performance is absolutely critical.

## Method

### Model

**Figure 4:**
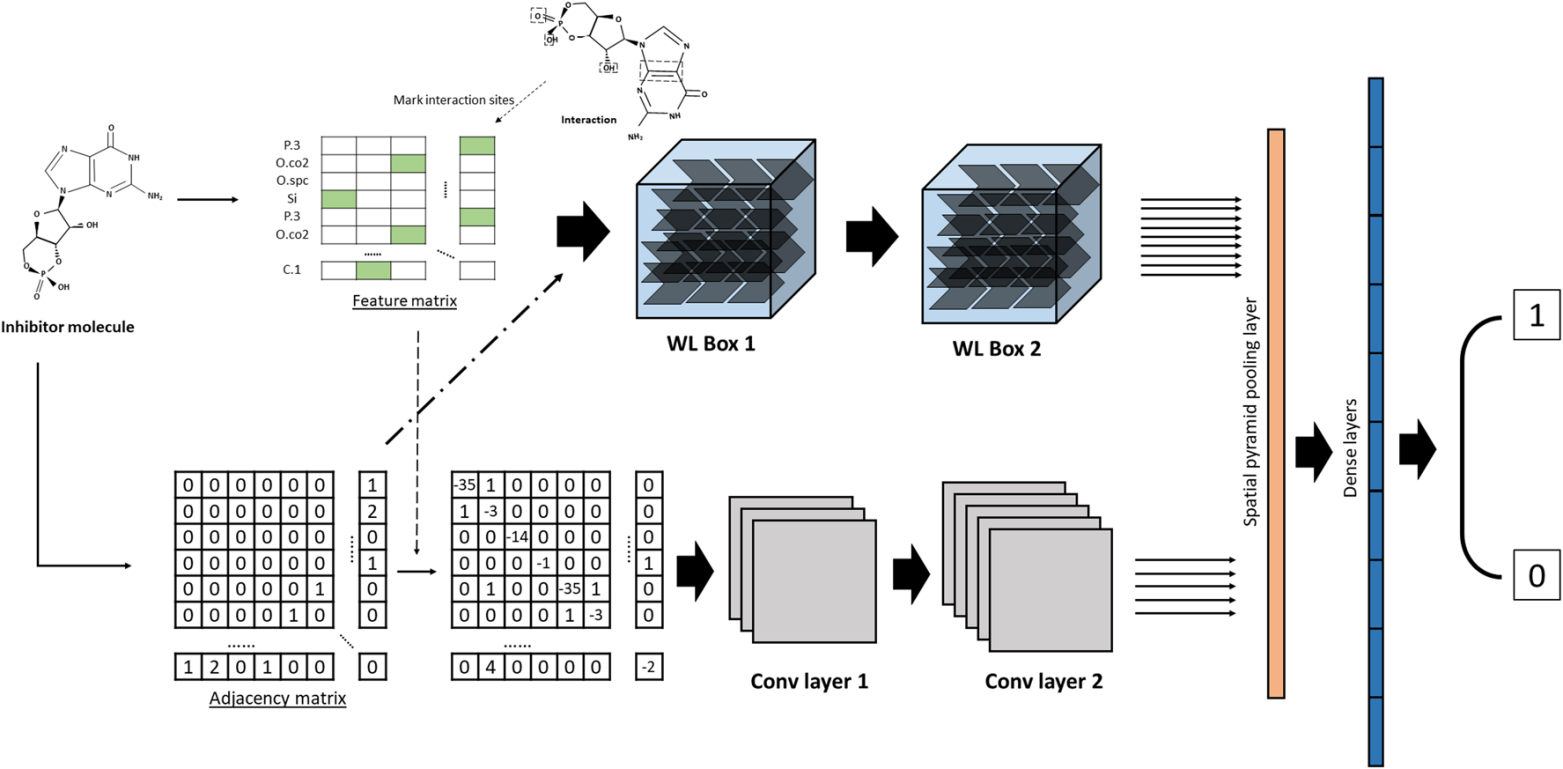
Architecture of the PISPKI model.

The architecture of the proposed PISPKI model is shown in Fig. 4. The model framework consists of four main parts: data preprocessing, WL Boxes, convolutional layers, and dense layers. First, each inhibitor molecule with *N* atoms is transformed to *N* pairs of feature matrices and structure adjacency matrices, where the *i*th atom is marked in the *i*th feature matrix to predict whether the corresponding atom is an interaction site. Note that the output of the model is assigned a value of 1 if the marked atom is predicted to be an interaction site. The feature matrices and structure adjacency matrices contain, respectively, atom and bond information of the molecules. Due to uncertainties about the number of atoms of the molecules, the sizes of the two matrices can be alterabled for different input data. Notably, zero-padding is not applied to satisfy all input data in the same size. After preprocessing, each pair of matrices is added to two submodules: WL Boxes and convolutional layers. The WL Boxes mainly process feature matrices using structure adjacency matrices as auxiliary information. Matrices with more significant features can be obtained from the output of WL Boxes; then the pooling layer processes new feature matrices into fixed-length vectors by applying the spatial pyramid pooling (SPP). By contrast, the convolutional layer processes structure adjacency matrices in which some atom information about the feature matrices is embedded in the diagonal elements, and the output is also processed by the SPP into a fixed-length vector. The resulting two vectors are concatenated as input to the dense layers for binary classification. Remarkably, the output vector from the pooling layer is obtained by combining the pooled results of the updated feature matrices and structure adjacency matrices, and the lengths of the vectors from the updated feature matrices are larger than those from the updated structure adjacency matrices, indicating that the output from the WL Boxes offers more information for the prediction of interaction sites by the classifier in the dense layer, thereby the WL Box module is the core of the PISPKI model.

### Preliminaries

Here, an inhibitor molecule is defined as an undirected graph denoted by *G*, which is represented by a 2-tuple (*F*, *S*), where *F* is a feature matrix representing the feature of the vertices and *S* is an adjacency matrix representing relationships among the vertices for *N* atoms and $N_e$ type bonds. Set $C=\{c^1,\ldots ,c^m,\ldots ,c^{N_c}\}$ consists of $N_c$ color types, where each color $c^m$ is represented by an $N_c$-dimensional binary vector. For each color vector $c^m=(c^m_1,c^m_2,\ldots ,c^m_i,\ldots ,c^m_{N_c})$, element $c^m_i$ is assigned a value of 1 if and only if $m=i$; otherwise the element is assigned a value 0.

Let *G*(*V*, *E*) be an undirected graph representing an inhibitor molecule, where $V = \{v_1,\ldots ,v_N\}$ is a set of atoms and *E* is a set of edges. Information on atoms (i.e., vertices in *G*) is represented by a binary feature matrix *F* of size $N \times N_c$ in which each row corresponds to an atom and the corresponding row vector is a color vector representing the atom type. Information on the edges of *G* is represented by an adjacency matrix *S* of size $N \times N$. $S_{ij}$ (i.e., the element of the *i*th row and *j*th column) is assigned a value of 0 if $\{v_i,v_j\} \notin E$, otherwise matrix $S_{ij}$ denotes the bond type (i.e., $S_{ij} \in \{1,2,\ldots ,N_e\}$).

Since graphs representing chemical structures are also considered, there is no self-loop; thus all diagonal elements of *S* are assigned a value 0. To effectively utilize the adjacency matrix, each vertex $v_i$ is assigned a label index $l_i$ according to the feature matrix row color $c^m$ by

$$\begin{aligned} l_i = m. \end{aligned}$$

Then, the convolutional layer input matrix $S_{conv}$ is obtained by the structure adjacency matrix *S* and the diagonal matrix as

$$\begin{aligned} S_{conv} = S-diag\left( l_1,l_2,\ldots ,l_N\right) . \end{aligned}$$

### Weisfeiler–Lehman algorithm

The Weisfeiler–Lehman (WL) algorithm, which was first proposed in 1968 to solve the *graph isomorphism problem*^35^, has recently been widely applied in neural network models. For every vertex $v_i$, features from neighboring vertices are aggregated and computed to update its own feature, which is computed as follows:

1 $$\begin{aligned} x'(v_i) = \mathcal {AGG}\left( x\left( v_i\right) ,emb\left\{ x\left( v_j\right) |v_j \in N\left( v_i\right) \right\} \right) , \end{aligned}$$

where $x(\cdot )$ and $x'(\cdot )$ are the original and updated features of vertices, respectively, and $N(v_i)$ denotes a set of neighboring vertices for vertex $v_i$, while *emb* is an embedding function based on neighborhood aggregation that concatenates features from neighboring vertices of $v_i$, and $\mathcal {AGG}$ is a custom function computing feature from the target vertex and its neighboring vertices. By implementing different functions, features can be updated in different ways. In addition, vertices can always have special features by several repetitions even with large graphs. Subsequently, the isomorphism of two graphs can be analyzed by examining the different features of updated set of vertices.

**Figure 5:**
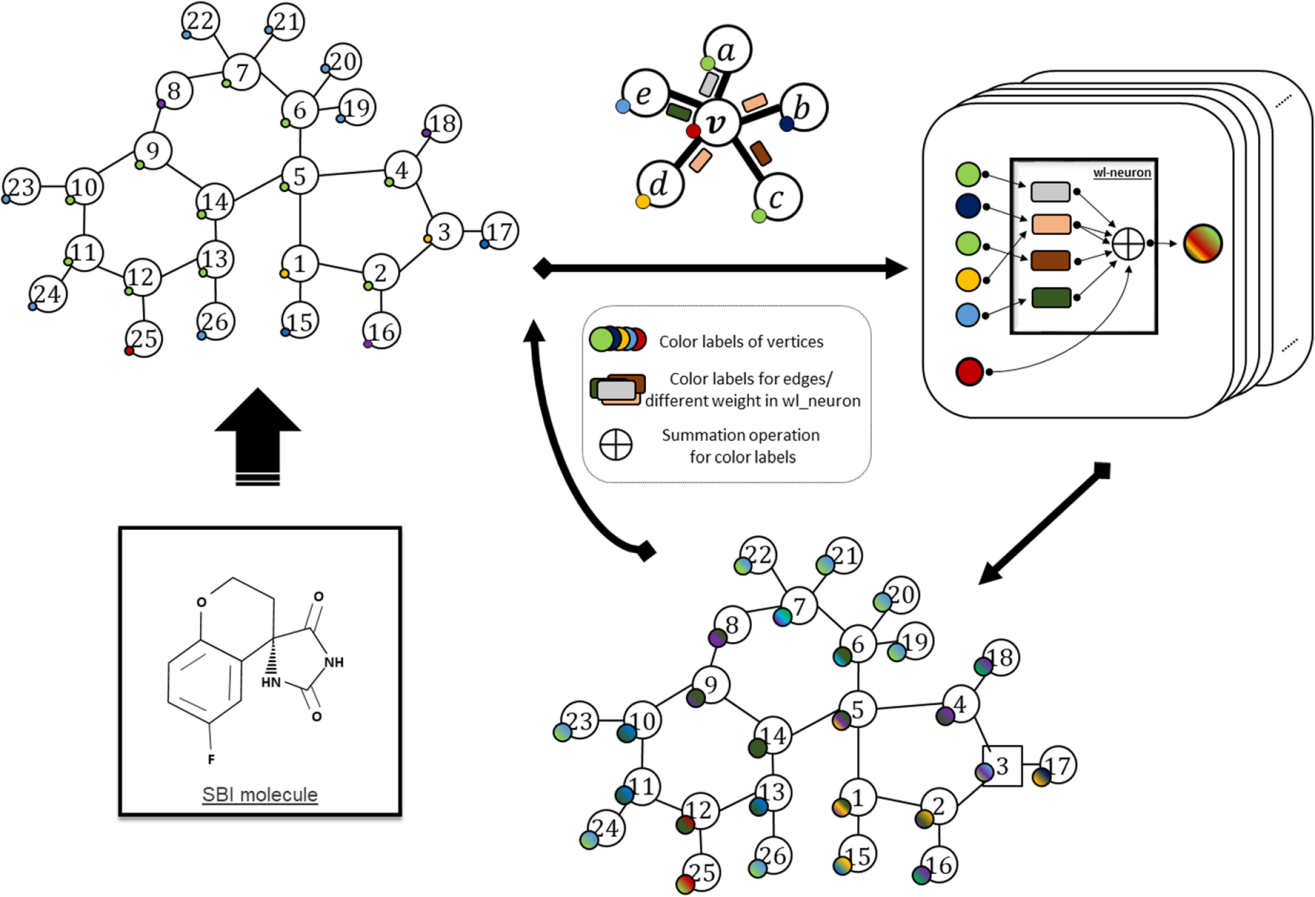
Weisfeiler–Lehman algorithm.

As mentioned in “Preliminaries” section, every vertex and edge have a *solid color* and label, respectively, and the *colors* of vertices are updated individually with the *colors* of the neighboring vertices and labels of the connected edge as illustrated in Fig. 5. The aggregation function is called *mix*, which can *blend* multiple colors together. Here, the *mix ratio* is dependent on the labels of the edges between the updated and neighboring vertices. After several repetitions of the algorithm, every vertex has a unique *blended color* as the feature in the inhibitor molecule graph.

### WL Box

**Figure 6:**
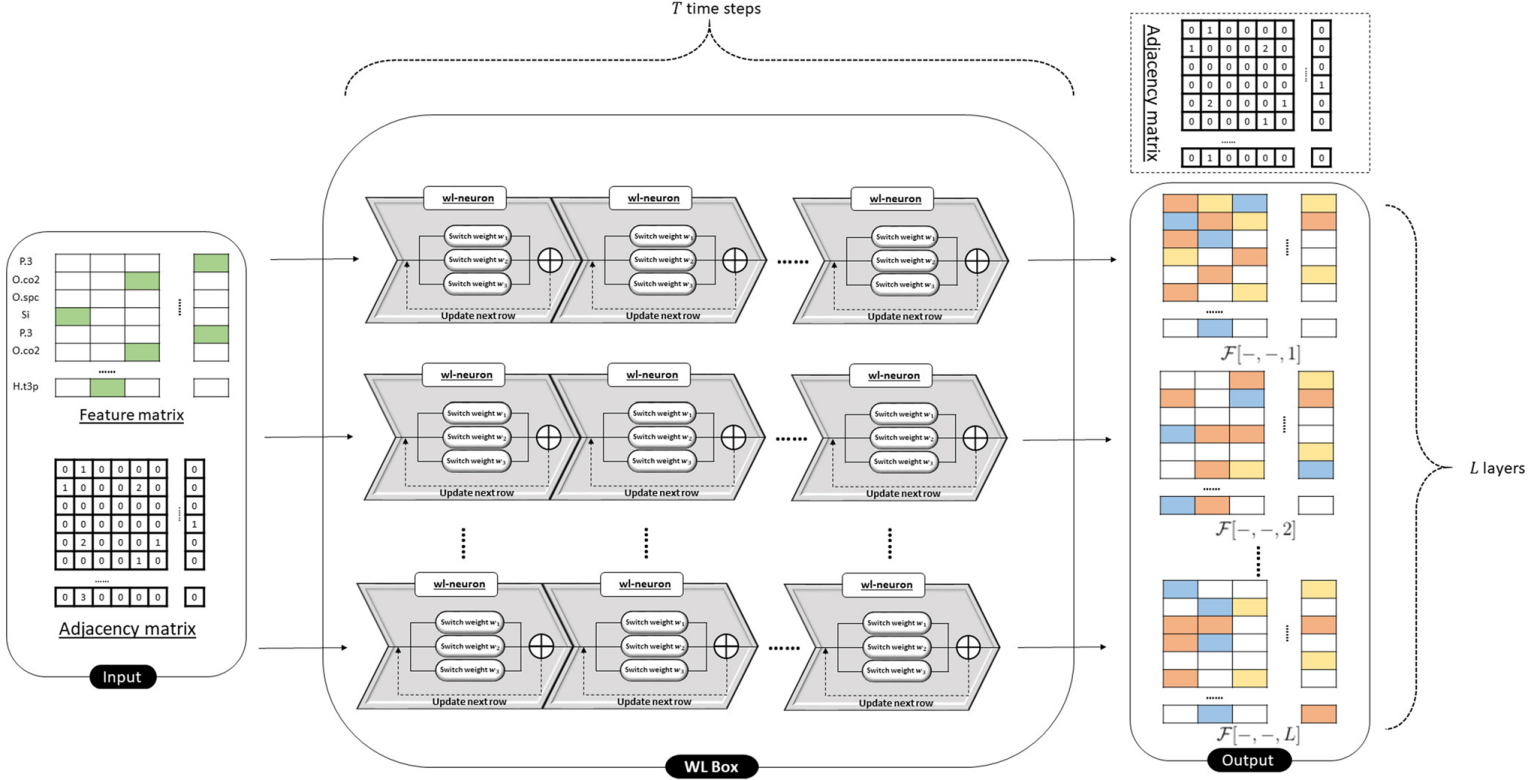
Structure of the WL Box.

The WL Box, which is the core of the PISPKI model, is based on the WL algorithm as described in “Weisfeiler-Lehman algorithm” section. A WL Box consists of $L\times T$ wl-neurons that are arranged by *L* layers and *T* time steps. Every layer contains *T* end-to-end wl-neurons and the feature matrix *F* is given an input for every first wl-neuron of the layers. Then, the wl-neuron updates the hidden state of the feature matrix in accordance with the WL algorithm and the structure adjacency matrix *S* is applied as supplementary information. The updated hidden state of the wl-neuron transfers to the next wl-neuron in each layer for $T-1$ time steps. The output of the last wl-neuron from the layer is an output of the WL Box. Hence, *L* new feature matrices can be obtained that contain more significant feature information in a WL Box. Furthermore, hidden states of feature matrices are updated in each layer individually, and there is no message exchange between layers in the box, as shown in Fig. 6.

The hidden state of a row is recurrently updated by

2 $$\begin{aligned} h^{(t)}_l(i)=h^{(t-1)}_l(i) + \sum \limits ^N_{j=1}w^{(t)}_{l,S_{ij}}h^{(t-1)}_l(j), \end{aligned}$$

where $h^{(t)}_l$ represents the current hidden state of the wl-neuron of the layer *l* at time step *t*, in which $l<L$ and $t<T$; $h^{(0)}_l$ denotes the initial state of the first neuron of the layer *l*, and $h^{(0)}_l = F$. Let $h^{(t)}_l=(h^{(t)}_l(1),h^{(t)}_l(2),\ldots ,h^{(t)}_l(N))$, and the *i*th row vector of $h^{(t)}_l$ is represented by $h^{(t)}_l(i)$; $w^{(t)}_{l,S_{ij}}$, which is a trainable *switch weight* (real number) depending on the layer, time step, and bond type $S_{ij}\in \{0,1,\ldots ,N_e\}$, where $w^{(t)}_{l,0}$ equals *zero* in any wl-neuron regardless of the layers and time steps.

Finally, the hidden states of the last neuron of each layer *l* at time step *T* are combined to a tensor ${\mathcal {F}}[i,j,l]$ as the output of a WL Box after processing with the activation function by

3 $$\begin{aligned} {\mathcal {F}}[i,j,l] = \sigma (h^{(T)}_l[i,j]), \end{aligned}$$

where ${\mathcal {F}}$ represents an output tensor of the WL Box, and ${\mathcal {F}}[-,-,l]$ denotes the *l*th *block* that is defined as a two-dimensional matrix consisting of all elements and the array from tensor ${\mathcal {F}}$ when the index is equal to *l* in *rank 3* in the tensor; $\sigma$ is an activation function, and $h^{(T)}_l$ is the last hidden state of the *l*th layer at time step *T*. Besides, the structure adjacency matrix *S* is invariant during the process in the WL Box and can be completely delivered to the next module if needed.

#### Multiple WL Boxes

Multiple WL Boxes can be implemented sequentially to further improve the feature matrix of the model. Two WL Boxes are assembled for the model as shown in Fig. 4. Notably, the second WL Box, which differs from the first WL Box, receives a tensor ${\mathcal {F}}$ as the input rather than matrix *F*, and so on. To get around this issue, superscripts are sequentially assigned to the feature tensor ${\mathcal {F}}$, such as ${\mathcal {F}}^{(1)},\ldots ,{\mathcal {F}}^{(M)}$ and the layer number *L*, such as $L^{(1)},\ldots ,L^{(M)}$, denoting the output tensor and maximum layer number of the first to the *M*th WL Box, respectively. The feature tensor of each WL Box is updated by

4 $$\begin{aligned} {\mathcal {F}}^{(m)} = \left\{ \begin{array}{ll} Concat_{l=1}^{L^{(m-1)}}( \Theta ({\mathcal {F}}^{(m-1)}[-,-,l])), &{}\quad m\ge 2 \\ \Theta (F), &{}\quad m=1 \end{array} \right. \end{aligned}$$

where ${\mathcal {F}}^{(m)}$ and ${\mathcal {F}}^{(m-1)}$ represent the output tensor of the *m*th and $m-1$th WL Box of the model, respectively; $\Theta$ denotes the WL update process function in a WL Box, as defined by Eqs. (2) and (3); ${\mathcal {F}}^{(m-1)}[-,-,l]\in {\mathbb {R}}^{N_c\times N}$ is the *l*th *block* of ${\mathcal {F}}^{(m-1)}$, and *F* is the model input feature matrix. Every *block* ${\mathcal {F}}^{(m-1)}[-,-,l]$ from tensor ${\mathcal {F}}^{(m-1)}$ is assigned to the *m*th WL Box as an individual input, and all $L^{(m-1)}$ output tensors are concatenated into one tensor ${\mathcal {F}}^{(m)}$ for the following computation operation.

### Spatial pyramid pooling

The spatial pyramid pooling (SPP) layer is applied to normalize the output from the WL Boxes and convolutional layers in this study, which is a novel and effective machine learning module proposed by He in 2015^40^. Different from classical pooling modules, SPP is a type of extensive research for region of interest operation, which further works with different sizes of pooling kernels in a matrix, and then concatenates the pooling results to a vector as the output. This also applies to hand-crafted pooling regions^41^ over scales of kernels that are dependent on different sizes of input matrices and adopts the spatial pyramid operation to obtain more comprehensive pooled feature maps, which are then converted to a fixed length vector.

A spatial pyramid consists of multiple stages, and each stage runs a pooling operation using the corresponding pooling coefficient. The notation *k* represents the pooling coefficient with $k=1,\ldots ,K$, where *K* is the stage number of a spatial pyramid. During each operation by the spatial pyramid, a hand-crafted kernel is applied, which yields precise $k\times k$ output from the inputted two-dimensional matrix. Due to differences in kernel size, each input is extended to

5 $$\begin{aligned} p^k_0 = \Xi _k(F), \end{aligned}$$

where *F* is a *block* of a tensor or matrix of $N_c\times N$ and $\Xi _k$ is the matrix extension function for pooling coefficient *k*. By applying the function, *F* is extended to a matrix $p^k_0 = {\mathbb {R}}^{k\cdot \lceil \frac{N_c}{k}\rceil \times k\cdot \lceil \frac{N}{k}\rceil }$, and all extended elements are equal to 0.

Here, the SPP layer receives two tensors ${\mathcal {F}}$ and ${\mathcal {S}}$ from the last WL Box and the convolutional layer. Two spatial pyramids are constructed (with stage numbers $K_F$ and $K_S$) to individually compute the two tensors. The pooling operation works with every *block* $K_F$ and $K_S$ times for the input tensors ${\mathcal {F}}$ and ${\mathcal {S}}$, respectively. Maximum SPP is applied to the output tensor ${\mathcal {F}}$ of the WL Box. For the *k*th stage of the spatial pyramid, each element of the *x*th column and *y*th row of matrix $p^k_f$ is computed by

6 $$\begin{aligned} p^k_f[x,y]=\sum \limits ^I_{i=1}\sum \limits ^J_{j=1}p^k_0[I\cdot (x-1)+i,J\cdot (y-1)+j]/IJ, \end{aligned}$$

where *k* denotes the pooling coefficient, $k\in \{1,\ldots ,K_F\}$; $p^k_0$ is an extended matrix computed by Eq. (5); $p^k_0[X,Y]$ denotes the element of the *X*th column and *Y*th row; (*I*, *J*) is the hand-crafted region in the *k*th stage, where $I=\lceil \frac{N_c}{k}\rceil$ and $J=\lceil {\frac{N}{k}}\rceil$.

Similarly, the average SPP was applied to the output tensor ${\mathcal {S}}$ from the convolutional layer. For the *k*th stage of the spatial pyrmaid, each element of the *x*th column and *y*th row of matrix $p^k_s$ is computed by

7 $$\begin{aligned} p^k_s[x,y]=\max \left( p^k_0[I\cdot (x-1):I,J\cdot (y-1):J]\right) , \end{aligned}$$

where *k* denotes the pooling coefficient, $k\in \{1,\ldots ,K_S\}$; $p^k_0$ is an extended matrix computed by Eq. (5), $p^k_0[X:I,Y:J]$ denotes a submatrix collecting elements from *X*th to $(X+I)$th columns and *Y*th to $(Y+J)$th rows; *I* and *J* constitute the hand-crafted region of the *k*th stage, where $I=\lceil \frac{N_c}{k}\rceil$ and $J=\lceil {\frac{N}{k}}\rceil$.

Finally, all elements are collated from the pooling matrices to an output vector as

8 $$\begin{aligned} P=Concat\left( \Phi (p^1_f),\Phi \left( p^2_f\right) ,\ldots , \Phi \left( p^{K_F}_f\right) ,\Phi \left( p^1_s\right) ,\ldots ,\Phi \left( p^{K_S}_s\right) \right) , \end{aligned}$$

where $\Phi$ is a function that converts a matrix to a vector, such as $\Phi (p^2_f)=(p^2_f[1,1],p^2_f[1,2],p^2_f[2,1],p^2_f[2,2])$.

Then, the output of the SPP layer is sent to a binary classifier for interaction site prediction by the dense layer.

## Supplementary information

Supplementary Information 1.
