# Supplementary material for: A novel graph convolutional neural network for predicting interaction sites on protein kinase inhibitors in phosphorylation
Source: Sci Rep. 2022 Jan 7;12:229. doi: 10.1038/s41598-021-04230-7 (PMC8742007; doi:10.1038/s41598-021-04230-7)
Supplement: Supplementary file 1 — Supplementary Information 1. [file 41598_2021_4230_MOESM1_ESM.pdf]

Supplementary Information

PISPKI: A graph convolutional neural network for predicting interaction sites on protein kinase inhibitors in phosphorylation

Feiqi WANG*
Bioinformatics Center, Insititute for Chemical Research,
Kyoto University, Gokasho, Uji, Kyoto, 611-0011, Japan.

Yun-Ti CHEN
Institute of Bioinformatics and Systems Biology,
National Yang Ming Chiao Tung University, Hsinchu, 300, Taiwan

Jinn-Moon YANG
Department of Biological Science and Technology,
National Yang Ming Chiao Tung University, Hsinchu, 300, Taiwan

Tatsuya AKUTSU*
Bioinformatics Center, Insititute for Chemical Research,
Kyoto University, Gokasho, Uji, Kyoto, 611-0011, Japan.

* Corresponding Authors

A Protein kinase classes & dataset quality

In this section, we show protein kinase classes and dataset qualities in the experiment.

**Kinase class**	**UniPort AC**
3-phosphoinositide-denpendent protein kinase	O15530
Aurora kinase	O14965
Circadian clock protein kinase	Q79PF4
Cyclin-denpendent kinase	P24931
Death-associated protein kinase	P53355
Dual specificity mitogen-activated protein kinase kinase	Q02750
Glucokinase	P35557
Glycogen synthase kinase	P49841
Serine/threonine-protein kinase	O96017<br>P51955<br>P11309<br>O14757
Tyrosine-protein kinase	O60674<br>P00519<br>P43405<br>P06239<br>P08631
Proto-oncogene tyrosine-protein kinase	P00523
Mitogen-activated protein kinase	P28482<br>Q16539<br>P47811<br>P53779

Table S1: Kinase classes

**Kinase class**	**Dataset quality** (p/n)
3-phosphoinositide-denpendent protein kinase	362/1060
Aurora kinase	273/1146
Circadian clock protein kinase	14/29
Cyclin-denpendent kinase	2521/7377
Death-associated protein kinase	98/253
Dual specificity mitogen-activated protein kinase kinase	337/596
Glucokinase	104/294
Glycogen synthase kinase	329/1038
Serine/threonine-protein kinase	1796/5654
Tyrosine-protein kinase	1003/3419
Proto-oncogene tyrosine-protein kinase	146/460
Mitogen-activated protein kinase	2826/7324

Table S2: Dataset quality

B Ablation experiment setting

The specific setting of ablation experiment is shown as follow:

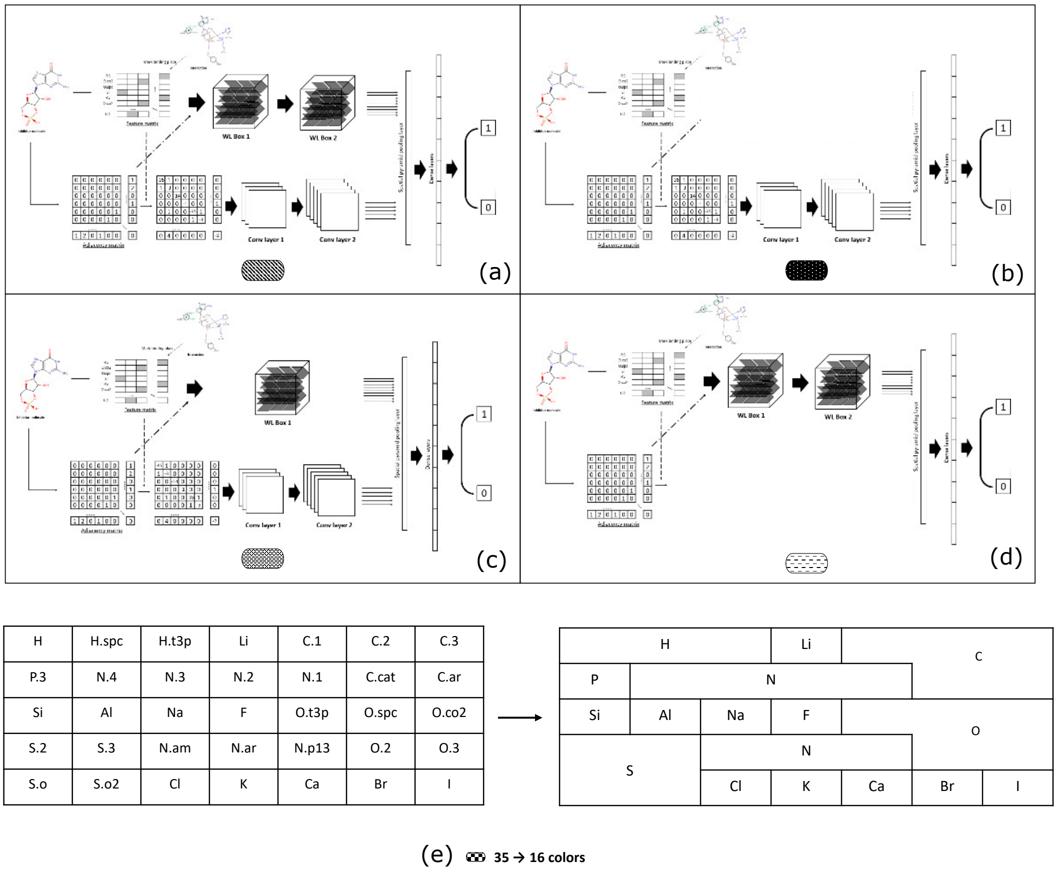

Full model Without two WL Boxes Only use one WL Box Without conv-layers 16 color

Figure S1: Ablation experiment setting
